# Off-target effects in CRISPR/Cas9 gene editing

**DOI:** 10.3389/fbioe.2023.1143157

**Published:** 2023-03-09

**Authors:** Congting Guo, Xiaoteng Ma, Fei Gao, Yuxuan Guo

**Affiliations:** ^1^ School of Basic Medical Sciences, Peking University Health Science Center, Beijing, China; ^2^ Peking University Institute of Cardiovascular Sciences, Beijing, China; ^3^ Department of Cardiology, Beijing Anzhen Hospital, Capital Medical University, Beijing, China; ^4^ Ministry of Education Key Laboratory of Molecular Cardiovascular Science, Beijing, China; ^5^ Beijing Key Laboratory of Cardiovascular Receptors Research, Beijing, China

**Keywords:** off-target effects, gene editing, CRISPR/Cas9, gene therapy, Cas9/sgRNA complex

## Abstract

Gene editing stands for the methods to precisely make changes to a specific nucleic acid sequence. With the recent development of the clustered regularly interspaced short palindromic repeats (CRISPR)/Cas9 system, gene editing has become efficient, convenient and programmable, leading to promising translational studies and clinical trials for both genetic and non-genetic diseases. A major concern in the applications of the CRISPR/Cas9 system is about its off-target effects, namely the deposition of unexpected, unwanted, or even adverse alterations to the genome. To date, many methods have been developed to nominate or detect the off-target sites of CRISPR/Cas9, which laid the basis for the successful upgrades of CRISPR/Cas9 derivatives with enhanced precision. In this review, we summarize these technological advancements and discuss about the current challenges in the management of off-target effects for future gene therapy.

## 1 Introduction

Genome editing tools hold great promise in treating genetic and non-genetic diseases. Early studies utilize zinc finger nucleases (ZFNs) and transcription activator-like effector nucleases (TALENs) for genome editing (Urnov et al., 2010; Joung and Sander, 2013; Shamshirgaran et al., 2022). ZFNs and TALENs depend on protein engineering of DNA-binding domains to recognize and edit specific DNA sequences. This engineering process could be ineffective, tedious and expensive, limiting the application of genome editing. The above problems were recently solved by the emergence of the clustered regularly interspaced short palindromic repeats (CRISPR)/Cas9 system. CRISPR/Cas9 is a class of ribonucleoprotein complexes formed by a Cas9 protein and a single guide RNA (sgRNA) (Jinek et al., 2012; Cho et al., 2013; Cong et al., 2013; Wang et al., 2016a). Cas9 can create DNA cleavage at desired genomic positions that are guided by precise base pairing between sgRNA and DNA, adjacent to a protospacer-adjacent motif (PAM) (Jinek et al., 2012; Cho et al., 2013; Cong et al., 2013; Wang et al., 2016a). Designing sgRNA is more convenient, programmable and cost-effective than designing DNA binding domains, thus CRISPR/Cas9 is more favored than ZFNs and TALENs and has revolutionized the biotechnology field (Zhang et al., 2021; Tran et al., 2022).

The Cas9/sgRNA complex produces site-specific DNA double-strand breaks (DSBs), stimulating homology-directed repair (HDR) or non-homologous end joining (NHEJ) pathways to achieve genome editing. HDR is an accurate but inefficient mechanism, which utilizes a homologous donor template to repair DNA cleavages (Li et al., 2019; Fu et al., 2021). By contrast, the error-prone NHEJ mechanism introduces small insertions and deletions (indels) and the exact sequence changes are unpredictable and uncontrollable (Fu et al., 2021). When the indels are deposited at the coding sequences of a given gene, NHEJ can cause frameshift mutations, resulting in non-sense-mediated mRNA decay and gene silencing (Wang et al., 2016a; Zischewski et al., 2017; Lindeboom et al., 2019).

Although CRISPR/Cas systems exhibit tremendous potential in translational medicine, off-target effects remain a major challenge (Fu et al., 2013; Hsu et al., 2013; Pacesa et al., 2022). The off-target effects occur when Cas9 acts on untargeted genomic sites and creates cleavages that may lead to adverse outcomes. The off-target sites are often sgRNA-dependent, since Cas9 is known to tolerate up to 3 mismatches between sgRNA and genomic DNA (Fu et al., 2013; Hsu et al., 2013; Wang et al., 2016a). In this scenario, *in silico* tools are useful to search for potential off-target sites in the whole genome and calculate the likelihood of an off-target editing (Naeem et al., 2020). Nevertheless, accumulative studies have proved that sgRNA-independent off-target effects also exist, urging unbiased experimental detection and validation (Richter et al., 2020; O'Geen et al., 2015). In this review, we summarize available methods for the assessment of off-target effects, indicating their advantages versus limitations. Some of these detection methods for off-targets prediction is applicable for other family of Cas nucleases, such as Cas12a (Cpf1), which also create DSBs on off-target sites (Kim et al., 2019). Furthermore, we discuss strategies to improve CRISPR/Cas9 specificity and to reduce undesired mutagenesis, which is crucial for their future application in gene therapy.

## 2 In silico prediction

CRISPR/Cas9 off-target effects can be predicted by *in silico* tools, which are usually open-source online software that can be conveniently accessed *via* internet (Bao et al., 2021). The prediction algorithms of these software are primarily based on sgRNA sequences, thus the outputs of these methods are usually biased toward sgRNA-dependent off-target effects. These computational methods usually insufficiently consider the complex intranuclear microenvironment such as the epigenetic and the chromatin organization states, thus off-target prediction by *in silico* tools needs further experimental validation (Table 1).

**TABLE 1:** In silico and experimental methods for genome-wide off-target prediction.

		Methods	Characteristics	Advantages	Disadvantages
In silico prediction	Alignment based models	CasOT [21]	Adjustable in PAM sequence and the mismatch number (at most 6)	Conveniently accessable *via* internet	Biased toward sgRNA-dependent off-target effects; results need experimental validation
		Cas-OFFinder [22]	Adjustable in sgRNA length, PAM type, and number of mismatches or bulges		
		FlashFry [23]	Provides information about GC contents		
		Crisflash [24]	High in speed		
	Scoring based models	MIT [15, 25]	Based on the position of the mismatches to the gRNA		
		CCTop [26]	Based on the distances of the mismatches to the PAM		
		CROP-IT [27]			
		CFD [28]	Based on a experimentally validated dataset		
		DeepCRISPR [29]	Considers both sequence and epigenetic feature		
		Elevation [30]			
Experimental detection	Cell-free methods	Digenome-seq [31–33]	Digests purified DNA with Cas9/gRNA RNP → WGS	Highly sensitive	Expensive; requires high sequencing coverage; requires a reference genome
		DIG-seq [34]	Uses cell-free chromatin with Digenome-seq pipeline	Concerning chromatin accessibility; higher validation rate than Digenome-seq	
		Extru-seq [35]	Pre-incubates live cells with Cas9/sgRNA RNP complex→rapidly kill cells by extruder→WGS	Low miss rate; low false positive rate	Expensive; difficult to detect Cas9-mediated large deletions, chromosomal depletions, and translocations
		SITE-seq [37]	A biochemical method with selective biotinylation and enrichment of fragments after Cas9/gRNA digestion	Minimal read depth; eliminated background; does not require a reference genome	Low sensitivity; low validation rate
		CIRCLE-seq [38–40]	Circularizes sheared genomic DNA→incubate with Cas9/gRNA RNP→linearized DNA for NGS		
	Cell culture-based methods	WGS [41–43]	Sequences the whole genome before and after gene editing	Comprehensive analysis of the whole genome	Expensive; limited number of clones can be analyzed
		ChIP-seq [44–47]	Analyzes binding sites of catalytically inactive dCas9	Detection of Cas9 binding sites genome-wide	Low validation rate; affected by antibody specificity and chromatin accessibility
		IDLV [48–52]	Integrates IDLV into DSBs	Detects off-targets in cells that are difficult to transfect	Low sensitivity; high false positive rate
		GUIDE-seq [36, 53–55]	Integrates dsODNs into DSBs	Highly sensitive, low in cost, low false positive rate	Limited by transfection efficiency
		LAM–HTGTS [57–59]	Detects DSB-caused chromosomal translocations by sequencing bait-prey DSB junctions	Accurately detects chromosomal translocations induced by DSBs	Only detects DSBs with translocation; efficiency limited by chromatin accessibility
		BLESS [60, 61]	Captures DSBs *in situ* by biotinylated adaptors	Directly capture DSBs *in situ*	Only identifies off-target sites at the time of detection
		BLISS [61, 62]	Captures DSBs *in situ* by dsODNs with T7 promoter sequence	Directly capture DSBs *in situ*; low-input needed	
	*In vivo* detection	Discover-seq [63]	Utilizes DNA repair protein MRE11 as bait to perform ChIP-seq	Highly sensitive; high precision in cells	Has false positives
		GUIDE-tag [64]	Uses biotin-dsDNA to mark DSBs	Highly sensitive; detects off target sites *in vivo*	The incorporation rate of biotin-dsDNA is relatively low (∼6%)

The off-target prediction software can be classified into two groups according to their output data format. The first group produces data describing the level of sgRNA alignment to the putative off-target sites in the genome. Representative software includes CasOT, Cas-OFFinder, FlashFry and Crisflash. CasOT is the first exhaustive tool to predict off-target sites in user-provided reference genome, and it allows custom adjustment of several parameters including the PAM sequence and the mismatch number (Xiao et al., 2014). Cas-OFFinder is more widely applicated due to its high tolerance of sgRNA length, PAM types, and the number of mismatches or bulges (Bae et al., 2014). FlashFry is designed for characterizing hundreds of thousands of CRISPR target sequences within short time. It is a high-throughput tool which can also provide information about GC contents and on/off-target scores (McKenna and Shendure, 2018). Crisflash is a tool for both sgRNA design and latent off-target discovery, which is over an order of magnitude faster than other software (Jacquin et al., 2019).

The second group of *in silico* tools can harness more complicated scoring models to facilitate computational nomination of the off-target sites. Such algorithm includes MIT score, CCTop (Consensus Constrained TOPology prediction), CROP-IT (CRISPR/Cas9 Off-target Prediction and I dentification Tool), CFD (Cutting frequency determination), DeepCRISPR and the Elevation software packages. The algorithm of MIT weights the position effect of the mismatches in the sgRNA to generate a score to evaluate off-target effects (Hsu et al., 2013; Haeussler et al., 2016). CCTop and CROP-IT generate scores based on the distances of the mismatches to the PAM (Singh et al., 2015; Stemmer et al., 2015). The CFD algorithm is derived from a CRISPR/Cas9 genetic screen experiment that assessed the off-target effects of thousands of sgRNAs (Doench et al., 2016). DeepCRISPR is a comprehensive computational platform which utilizes deep learning to predict off-target cleavage sites. It considers epigenetic features such as chromatin opening and DNA methylation to figure out genome-wide off-target profiles (Chuai et al., 2018). Similarly, the Elevation tool also includes DNA accessibility information to predict potential off-target sites (Listgarten et al., 2018). The disadvantage of Elevation is it only works with human exome (GRCh38), limiting its broader usage in other organisms (Listgarten et al., 2018).

## 3 Experimental detection

### 3.1 Cell-free methods

Cell-free methods reconstitute nuclease reaction on DNA or chromatin that are extracted from the cells to directly identify genomic cleavages in the test tubes. Representative cell-independent methods are Digenome-seq (digested genome sequencing), DIG-seq (Digenome-seq using cell-free chromatin DNA), Extru-seq, SITE-seq (selective enrichment and identification of tagged genomic DNA ends by sequencing) and CIRCLE-seq (circularization for *in vitro* reporting of cleavage effects by sequencing) (Table 1).

**FIGURE 1:**
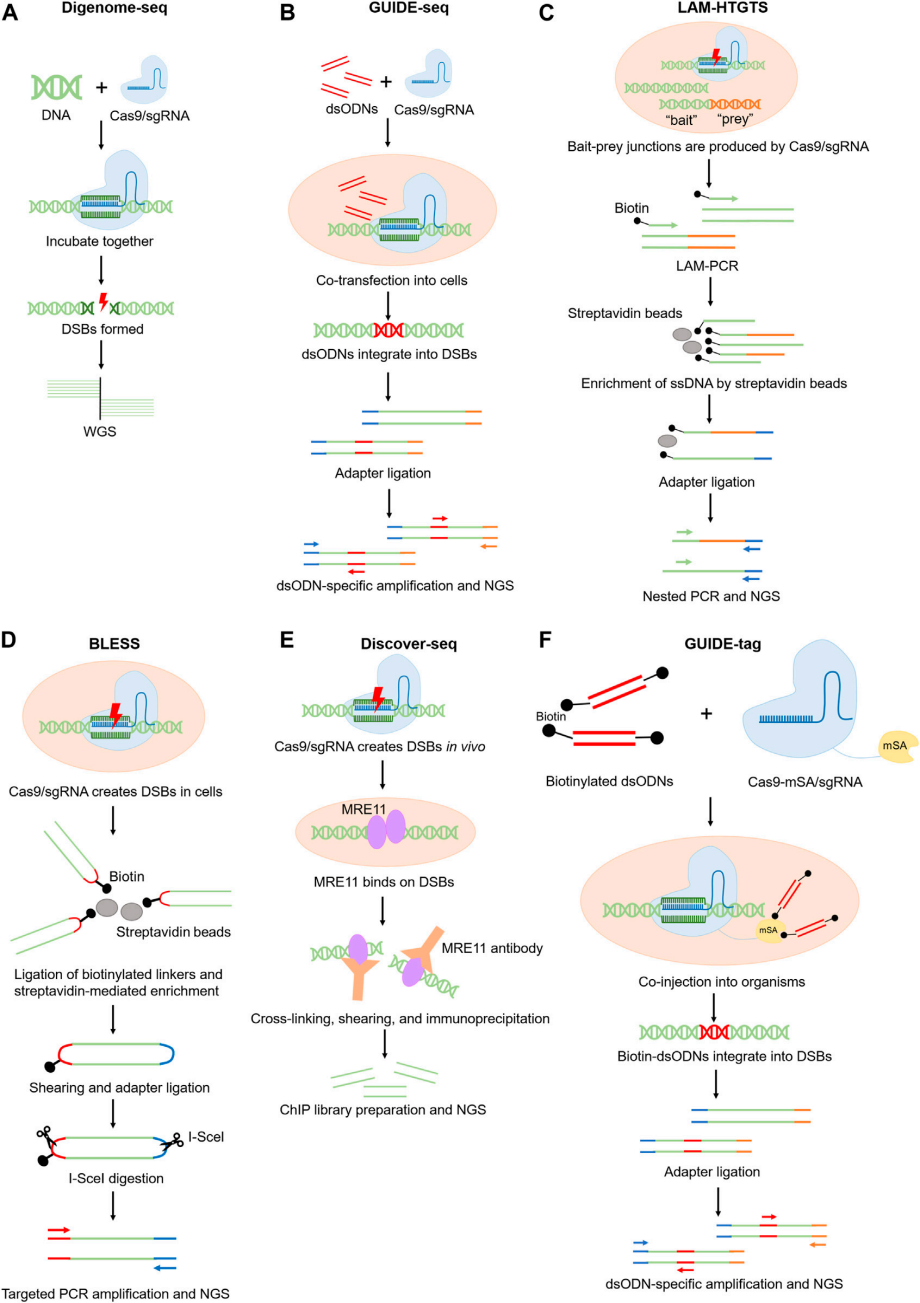
Schematics of experimental methods for genome-wide off-target prediction.

Digenome-seq is a highly sensitive method that can identify indels with 0.1% frequency or lower (Kim et al., 2015). In this method, genomic DNA is firstly extracted from cells and incubated with Cas9/sgRNA ribonucleoprotein (RNP) complex for gene editing. The edited DNA is next analyzed by whole-genome sequencing (WGS) to detect sequences accurately sharing one end, which indicates the loci where DSBs exist. The current Digenome-seq pipeline is equipped with a refined scoring algorithm and allows off-target sites screening involving multiple sgRNA (Kim et al., 2016). Due to the high background of non-specific DSBs in the purified DNA samples, Digenome-seq requires high sequencing coverage (∼400–500 million reads for human genome) thus the sequencing cost can be relatively high (Kim et al., 2019). The demand for a high-quality reference genome also limits its broader use in uncommon organisms (Figure 1A).

A major caveat of Digenome-seq is that the chromatin states are omitted in the assessment of the off-target effects. To solve this problem, an updated version of Digenome-seq called DIG-seq (Kim and Kim, 2018) was developed. DIG-seq applies cell-free chromatin instead of purified DNA to the Digenome-seq pipeline, demonstrating a higher accuracy in nominating off-target sites. The comparative study between Digenome-seq and DIG-seq strongly indicated the influence of chromatin states on the off-target activity (Kim and Kim, 2018).

To further retain the genome near its intracellular state for better off-target detection, Jeonghun et al. recently reported Extru-seq (Kwon et al., 2023). In this method, suspended live cells are firstly mixed with purified Cas9/sgRNA RNP complex. Then the cells are mechanically lysed by passing through pores smaller than the cell diameter to release genomic DNA reacting with Cas9/sgRNA before WGS (Kwon et al., 2023). Compared to other cell-free methods such as Digenome-seq, the false positive rate of Extru-seq is significantly lower (Kwon et al., 2023). Interestingly, compared to cell-based methods such as GUIDE-seq (see next section) (Tsai et al., 2015), Extru-seq exhibited much lower false negative rate (2.3% versus 29%) (Kwon et al., 2023). Thus, Extru-seq integrates advantages of both cell-free and cell-based methods.

All above methods involve the expensive WGS step. To reduce such cost, scientists developed SITE-seq, a method that adds a selective biotinylation reaction on the cleaved genomic sites and leverages streptavidin pulldown to enrich these sites before sequencing (Cameron et al., 2017). SITE-seq reduces the background noise of Digenome-seq and requires much less sequencing coverage (∼0.62–2.46 million reads for human genome). Nevertheless, the accuracy of SITE-seq in finding off-target sites is still low, with only 10% positive hits that could be validated by targeted sequencing (Kim et al., 2019).

CIRCLE-seq is another method that can detect genome-wide off-target sites without performing WGS. In this method, genomic DNA is first sheared and circularized by intramolecular ligation. With the presence of Cas9/sgRNA complexes, the circular DNA fragments were selectively linearized upon Cas9 nuclease cleavage before they become available for library construction and high-throughput sequencing (Tsai et al., 2017; Lv et al., 2022; Pan et al., 2022). In this method, non-specific linear DNA and undigested circular DNA can be efficiently removed, greatly reducing the background in off-target detection. CIRCLE-seq demands only ∼4–5 million reads for a successful analysis for human genome (Kim et al., 2019). However, the false positive rate of CIRCLE-seq is still high and needs careful downstream validation by targeted sequencing (Tsai et al., 2017).

### 3.2 Cell culture-based methods

Because the intranuclear context influences the behavior of the genome editors, direct assessment of the off-target effects in cells would be more favorable than cell-free methods. Currently, WGS, Cas9 ChIP–seq (chromatin immunoprecipitation followed by high-throughput sequencing), IDLVs (integrase defective lentiviral vectors), GUIDE–seq (genome-wide, unbiased identification of DSBs enabled by sequencing), LAM-HTGTS (linear amplification-mediated high-throughput genome-wide sequencing), BLESS (breaks labeling, enrichment on streptavidin, and next-generation sequencing), and BLISS (breaks labeling *in situ* and sequencing) have been developed to achieve this goal (Table 1).

WGS analysis of off-target effects has been well documented in cell culture studies (Smith et al., 2014; Veres et al., 2014; Iyer et al., 2015). By comparing the genome sequences before and after CRISPR/Cas9 editing, WGS can directly uncover desired and unwanted editing events. The accuracy and sensitivity of WGS in off-target detection is determined by sequencing depth, thus when there is a demand to determine low-frequency off-target sites, WGS would become expensive.

To avoid the prohibitive cost of WGS, CRISPR/Cas edited sites need to be enriched before sequencing. One such approach involves Cas9 ChIP-Seq to determine the binding sites of Cas9 on the genome. This method uses catalytically inactive Cas9 (dCas9), which can bind to genome DNA without introducing DSBs and detaching from the edited sites (Kuscu et al., 2014; Wu et al., 2014). One study reported dCas9 ChIP-Seq with 12 different sgRNAs in HEK293T cells and successfully validated DNA cleavage at about 50% of the predicted off-target sites (Kuscu et al., 2014). However, other publications observed a much lower validation rate (Cencic et al., 2014; Duan et al., 2014; Tsai et al., 2015). These studies indicated that Cas9 could associate with genomic loci without exerting its nuclease function, deposing a major caveat in Cas9 ChIP-Seq-based off-target detection assay.

Intracellular labeling of CRISPR/Cas9-edited loci for the enrichment of these DNA fragments is necessary for more precise determination of the off-target effects in cells. One such labeling tool is called the integrase-defective lentiviral vectors (IDLV), which displays the propensity to integrate into the vicinity of DSBs (Wang et al., 2015; Kim et al., 2019). IDLV was first designed to detect DSBs created by ZFNs and TALENs (Gabriel et al., 2011; Wang et al., 2015; Wang et al., 2021). The IDLV-integrated sites can be selectively amplified by PCR for high-throughput sequencing. Empowered by the robust transduction ability of lentivirus in certain cell types, this method can detect off-target effects in cell types that are otherwise difficult to transfect (Ferrari et al., 2022). The disadvantages of IDLV include its low sensitivity and high false positive rate, probably due to non-specific IDLV integration and PCR amplification (Martin et al., 2016).

Another more popular method to detect off-target sites in cells is called GUIDE-seq (Tsai et al., 2015). This technique relies on the delivery of double-stranded oligonucleotides (dsODNs) with known sequences, which can integrate into DSBs during NHEJ (non-homologous end joining). The integrated dsODNs provide templates for targeted PCR amplification and sequencing of the tagged DNA fragments (Tsai et al., 2015; Malinin et al., 2021; Yaish et al., 2022) (Figure 1B). GUIDE-seq can detect off-target sites with indel frequencies as low as 0.03% (Tsai et al., 2015). GUIDE-seq is more sensitive than the IDLV method because dsODNs integrate more efficiently and precisely into DSBs, while the integration events of IDLV is low in number and can distribute as far as 500bp away from the actual DSB sites (Tsai et al., 2015; Cromer et al., 2023). A primary limitation of GUIDE-seq is relevant to the low delivery efficiency of dsODNs into cells, which results in detection of only 30%–50% of all the DSBs (Tsai et al., 2015; Pan et al., 2022).

Both IDLV and GUIDE-seq rely on the DNA insertion activity during NHEJ. However, DSBs can also lead to chromosome translocation and rearrangement. To better detect such DSBs, LAM–HTGTS was developed (Frock et al., 2015; Hu et al., 2016). In this technique, mammalian cells are cultured with Cas9 nuclease to create “bait” and “prey” DSBs. The “bait” DSBs are the sites that are previously known to be cleaved by the nuclease, while the “prey” DSBs are the unknown off-target sites that are expected to ligate with the “bait” site after chromosome rearrangement. The bait-prey junctions can be linearly amplified and enriched using a biotinylated primer. Then these DNA are ligated to adaptors and selectively amplified by nested PCR for NGS analysis (Schmidt et al., 2007; Hu et al., 2016) (Figure 1C). The advantage of this method is the ability to detect chromosomal translocations that may be missed by other methods (Hu et al., 2016; Li et al., 2019; Xu et al., 2023). However, because DNA translocation occurs less frequently than small DNA insertions, whether the sensitivity of LAM–HTGTS is comparable to other off-target detection methods remains questionable (Hu et al., 2016; Li et al., 2019).

IDLV, GUIDE-seq and LAM–HTGTS measure the DSB-derived DNA fragments to indirectly infer the presence of DSBs. Alternatively, DSB can be directly detected by BLESS (direct *in situ* breaks labeling, enrichment on streptavidin and next-generation sequencing), which captures DSBs *in situ via* the ligation of biotinylated linkers to cleavage sites in fixed cells (Crosetto et al., 2013) (Figure 1D). BLESS demonstrates a false positive rate lower than 1% (Crosetto et al., 2013; Yan et al., 2017; Kim et al., 2019), validating the accuracy of this method. The predominant limitation of this technique is that BLESS only captures DSBs that are present at the moment of sample fixation, which underrepresents the off-target events. Therefore, BLESS demands millions of cells to reduce the false negative rate.

To enhance the sensitivity of BLESS and reduce its requirement on cell number, BLISS (breaks labeling *in situ* and sequencing) technology was developed (Yan et al., 2017). BLISS ligates DSB ends with adapters containing the T7 promoter, so the tagged DNA fragments can be linearly amplified *via* T7-mediated transcription before sequencing (Yan et al., 2017; Ballarino et al., 2021). Compared to BLESS, BLISS demands only a few thousand cells and demonstrates a higher sensitivity. For example, Winston et al. performed side-by-side comparison between BLISS and BLESS to detect the off-target sites of validated sgRNAs targeting EMX1 or VEGFA (Yan et al., 2017). For the sgRNA targeting EMX1, BLESS uncovered 6 off-target sites, all of which are included in the 10 genuine off-target sites that BLISS discovered. Similarly, for the sgRNA targeting VEGFA, besides the 16 off-target sites that were detected by both methods, BLISS identified 27 additional off-target sites that are not found by BLESS (Yan et al., 2017). Thus the sensitivity of BLISS is more than two folds higher than BLESS.

### 3.3 *In vivo* detection

A major application of the CRISPR/Cas9 system is to edit somatic cells for *in vivo* gene therapy. Methods to directly measure the off-target effects in tissues and even in living organisms would be critical to fully assess the safety of gene editing drugs. Exemplary techniques include Discover–seq (discovery of *in situ* Cas off-targets and verification by sequencing) and GUIDE-tag (Table 1).

Discover-seq utilizes MRE11, an endogenous DNA repair protein, to identify CRISPR-Cas-induced DSBs *in vivo* (Wienert et al., 2019). During DNA damage responses, MRE11 specifically docks on DSBs, which can be detected by MRE11 ChIP-seq. Because good ChIP-grade MRE11 antibodies are available and there is no need to deliver any exogenous components to the body, Discover-seq is broadly applicable for various types of tissue or cell samples (Wienert et al., 2019; Cromer et al., 2023). However, because MRE11-DSB binding is transient, Discover-seq only captures DSBs that are present at the moment of sample preparation (Wienert et al., 2019). Therefore, the sensitivity of Discover-seq should be carefully assessed, concerning a potentially high false negative rate. Currently there is no standardized approach to confirm the sensitivity of Discover-Seq. A potential solution is to use multiple orthogonal approaches to cross-validate the false negative results to ensure the sensitivity of Discover-Seq is sufficient for the given application (Figure 1E).

GUIDE-tag is a more recently developed method to detect off-target effects in cell culture and in tissues. GUIDE-tag was derived from GUIDE-seq but with an improved dsODN capture system to increase the discovery rate of DSBs (Liang et al., 2022). More specifically, a monomeric streptavidin (mSA) is fused to the Cas9 nuclease to form a Cas9-mSA/sgRNA ribonucleoprotein complex in GUIDE-tag. During genome editing, mSA recruits biotinylated dsODN to facilitate its integration into DSBs *via* NHEJ (Liang et al., 2022) (Figure 1F). To compare the sensitivity of GUIDE-tag versus Discover-seq, Shun-Qing et al. have evaluated *in vivo* GUIDE-tag in mouse liver at a target site in *Pcsk9* that has been previously characterized by Discover-seq. Among the 26 off-target sites that were originally detected by Discover-seq, GUIDE-tag successfully captures 24. In addition, GUIDE-tag uncovers 16 new off-target sites that were not discovered by Discover-seq (Liang et al., 2022). Thus GUIDE-tag is a more sensitive method than Discover-seq.

## 4 Strategies to reduce off-target effects

### 4.1 Cas9 improvement

Studies on the mechanisms by which the prototypic SpCas9 (*Streptococcus pyogenes* Cas9) functions have provided critical insights about the structural basis of off-target effects (Hsu et al., 2013). Based on this information, scientists proposed that the fidelity of SpCas9 can be enhanced by reducing non-specific Cas9/sgRNA binding to DNA, particularly the non-targeted DNA strand. This idea led to the rational design of SpCas9 mutants such as enhanced SpCas9 (eSpCas9) and SpCas9-HF1 (HF1 for high-fidelity variant #1). Further protein structure analysis of eSpCas9 and SpCas9-HF1 revealed the presence of a proof-reading mechanism that trapped these mutants in an inactive state when bound to mismatched targets. Accordingly, scientists further designed hypaCas9 (hyper-accurate Cas9) (Chen et al., 2017), which demonstrates higher on-target activity and lower off-target effects than eSpCas9 and SpCas9-HF1 (Kleinstiver et al., 2016; Slaymaker et al., 2016; Chen et al., 2017).

To compare the fidelity of the above Cas9 derivatives that were developed by rational engineering, scientists compared both the activity of these mutants at on-target sites as well as the number of detectable off-target sites using previously reported sgRNAs. Janice S et al. shows that eSpCas9, SpCas9-HF1 and hypaCas9 maintained high on-target activity (>70% of wildtype SpCas9) at 23/24, 18/24, and 19/24 tested sites, respectively. In addition, GUIDE-seq results demonstrated dramatically decreased number of off-target sites. For example, among the 134 off-target sites of one sgRNA targeting VEGFA with SpCas9, only 19, 24, and 18 of the off-target sites are also detectable with eSpCas9, SpCas9-HF1 and hypaCas9, respectively (Chen et al., 2017).

SpCas9 variants with enhanced specificity can also be developed by an unbiased high-throughput screen. A good example is a yeast-based screen for SpCas9 mutants with random mutations in the REC3 (REC stands for recognition) domain, the critical Cas9 domain that is responsible for the pairing between the genomic DNA and the Cas9/sgRNA complex. This screen identified four key beneficial point mutations in SpCas9, giving rise to evoCas9, a variant that exhibits fidelity exceeding SpCas9-HF1 and eSpCas9 (Kleinstiver et al., 2015; Casini et al., 2018). Antonio et al. performed GUIDE-seq to directly compare the off-target effects of evoCas9, SpCas9-HF1 and eSpCas9 relative to wildtype SpCas9. They found that the number of off-target sites reduced by 98.7%, 95.4% and 94.1%, respectively. Meanwhile, the absolute on-target activity of these Cas9 mutants are not dramatically reduced as compared to SpCas9 (Casini et al., 2018).

In addition to protein engineering, the specificity of CRISPR/Cas9 editing can also be enhanced with paired SpCas9 nickases (Frock et al., 2015). Cas9 nickases are mutated Cas9 nucleases that cut only one strand of the DNA. When two sgRNAs are designed to simultaneously cut the two opposite strands of DNA within a small distance, DSBs can be created with markedly lower off-target effects (Hsu et al., 2013; Frock et al., 2015). A major caveat of this strategy lies in the difficulty to identify the two properly positioned sgRNAs on the targeted genomic site, given the restriction of PAM sequences.

The off-target effect can also be reduced by discovering new Cas9 homologs that use rarer PAM sequences, thereby exhibiting less probability to dock on non-targeted genomic DNA. For example, in contrast to SpCas9, which uses a relatively common 5’-NGG-3’ PAM, the SaCas9 that is derived from *Staphylococcus aureus* requires a more complicated PAM sequence of 5’-NGGRRT-3’ (Kumar et al., 2018). Similarly, St1Cas9 and St3Cas9 from *Streptococcus thermophilus* recognize longer PAM sequences, which are 5’-NNAGAAW-3’ and 5’-NGGNG-3’, respectively (Muller et al., 2016). In addition to the enhanced specificity, these Cas9 homologs also provide the opportunity to target genomic sites that are otherwise not editable by SpCas9. However, it should be mentioned that using a Cas9 homolog with a rare PAM has the trade-off of many sequences no longer being targetable (Table 2).

**TABLE 2:** Strategies to reduce off-target effects.

	Classification	Description	Effect
Cas9 improvement	SpCas9 variants [65–69]	SpCas9-HF1, eSpCas9, hypaCas9: rational designed SpCas9 mutants	Maintained or increased on-target efficiency and decreased off-target effects
		EvoCas9: unbiased high-throughput screen	
		Paired SpCas9 nickase: mutate at RuvC or HNH domain	
	Cas9 orthologous [70–71]	SaCas9: from *Staphylococcus aureus*; (PAM: 5’-NGGRRT-3’)	Complex PAM sequence
		St1Cas9, St3Cas9: From *Streptococcus* thermophilus; (PAM: 5’-NNAGAAW-3’ and 5’-NGGNG-3’)	
gRNA improvement	gRNA length adjustment [72–75]	Extended gRNA: GGX20	Decreased off-target effects
		Truncated gRNA: truncated by 2-3bp at 5’ end	
	Chemical modifications [76–78]	Incorporation of MP/bridged nucleic acids/locked nucleic acids	Increased on-target efficiency and decreased off-target effects
		Replacement of RNA nucleotides into DNA nucleotides	
Delivery methods improvement	Application in cell culture [89–94]	Plasmid transfection	May cause accumulation of off-target mutations
		Viral transduction	
		RNP electroporation: Rapid transfection and turnover	Eliminate off-target effects
	Application *in vivo* [95–105]	AAV: Last for years in terminally differentiated cell types	May cause accumulation of off-target mutations
		LNP: Can be quickly degraded *in vivo*	Eliminate off-target effects

### 4.2 sgRNA improvement

In addition to the Cas9 protein, sgRNA can also be engineered to enhance genome editing fidelity. Primarily, the sequence of sgRNAs is a crucial factor affecting on-target and off target efficiency. Different sgRNAs targeting the same gene locus can have distinct outcomes, thus it is important to screen for a suitable sgRNA for the interested gene locus before further experiment (Doench et al., 2016). Apart from the impact of sgRNA sequences, accumulative evidence showed that the specificity of Cas9 activity can be enhanced by extending or truncating sgRNAs (Cho et al., 2014; Fu et al., 2014; Kim et al., 2016; Kim et al., 2017; Liang et al., 2019). In the sgRNA extending approach, two guanine nucleotides are usually added at the 5’ end of sgRNAs (termed 5’-GGX20) (Cho et al., 2014). These extra guanine nucleotides are favored in T7-promoter driven transcription, and may hinder the interaction between the Cas9/sgRNA complex and the DNA at the off-target sites (Cho et al., 2014; Kim et al., 2019). On the other hand, sgRNA truncation by 2-3bp at 5’ end is also reported to decrease off-target effects while maintaining on-target editing efficiency (Fu et al., 2014). Even more reduction of undesired mutagenesis is achieved when truncated sgRNAs are applied to paired Cas9 nickases (Cho et al., 2014).

Apart from sgRNA length adjustment, chemical modifications on sgRNAs can also influence their off-target effect. One study incorporated 2’-O-methyl-3’-phosphonoacetate (MP) into the ribose-phosphate backbone of sgRNA and found that MP modifications at certain positions can enhance on-target specificity while dramatically reducing off-target activity (Ryan et al., 2018). Incorporation of bridged nucleic acids (2’,4’-BNA^NC^ [N-Me]) or locked nucleic acids (LNA) into the sgRNAs also reduces the kinetics of Cas9 nuclease reactivity, thereby improving the specificity of genome editing (Cromwell et al., 2018). The replacement of ribonucleotides by deoxyribonucleotides also reduced off-target effects (Yin et al., 2018). The major caveat of sgRNA modification strategies is that they are currently restricted to genome editing applications that use synthetic RNAs but not DNA-based transgenes (Table 2).

### 4.3 DSB-independent gene editing

Cas9-mediated DSB generation is the main source of CRISPR/Cas9 off-target effect. Therefore the new versions of gene editors that do not create DSBs usually exhibit greater specificity of genome editing. Base editors are one such tool, which couple Cas9 nickases with nucleotide deaminases to achieve single nucleotide conversion without introducing DSBs. The most popular base editors are the adenine base editor (ABE) and the cytosine base editor (CBE). ABE catalyzes the editing from adenine to guanine and CBE converts cytosine to thymine (Komor et al., 2016; Gaudelli et al., 2017; Wu et al., 2022).

Although CBE and ABE greatly reduce the classic off-target effects of CRISPR/Cas9 systems, they create new formats of off-target effects such as RNA editing and sgRNA-independent DNA editing (Grünewald et al., 2019; Jin et al., 2019; Zhou et al., 2019; Zuo et al., 2019). These effects are likely introduced by the excessive deaminase activity that are not restricted by the Cas9/sgRNA binding to the corresponding genomic loci. The RNA off-target effects can be detected by RNA-seq (Grünewald et al., 2019; Zhou et al., 2019) and are believed to be transient given the short lifetime of RNA. The sgRNA-independent DNA editing is likely deposited on loci where DNA unwinds into an R-loop so single-strand genomic DNA is exposed to deaminases (Jin et al., 2020; Richter et al., 2020).

The recent development of EndoV-seq and Detect-seq have greatly facilitated the detection of off-target effects of ABE and CBE on genomic DNA. EndoV-seq leverages the endonuclease V (EndoV) to specifically cut inosine in DNA (Liang et al., 2019). Since inosine is the nucleoside intermediate that is created by ABE, EndoV can specifically cut DNA loci that are edited by ABE. Such DNA cuts can be detected by sequencing using an experimental platform similar to Digenome-seq (Liang et al., 2019). Similar to EndoV-seq, Detect-seq measures CBE off-target effects by tracking its reaction intermediates (Lei et al., 2021). More specifically, Detect-seq chemically labels deoxyuridine, the deamination product of cytosine, and enriches CBE edited genomic loci by biotin-streptavidin pulldown for deep sequencing analysis (Lei et al., 2021).

In addition to base editors, CRISPR/Cas9-mediated off-target effects can also be reduced by epigenetic editors (Willyard, 2017). These tools utilize enzymatically dead Cas9 (dCas9) to direct epigenetic modulation on targeted genes, thereby altering their endogenous expression level. The advantage of epigenetic editors is they completely avoid the permanent edits on DNA. However, epigenetic editors could potentially exert off-target effects at the epigenome level (Willyard, 2017), which should be carefully evaluated by ChIP-seq-based analysis.

### 4.4 Delivery method improvement

The activity and fidelity of gene editing is heavily affected by the expression level and duration of the editors in the cells. Therefore, methods to deliver Cas9/sgRNA into the target cells profoundly influence its off-target effect (Lohia et al., 2022; Taha et al., 2022). In cell culture applications, Cas9/sgRNA can be delivered *via* plasmid transfection, ribonucleoprotein (purified Cas9/sgRNA complex, RNP) electroporation, or viral transduction. RNP electroporation exhibits higher on-target editing efficiency and lower off-target mutations than the other delivery methods (Kim et al., 2014; Ramakrishna et al., 2014). Similar to RNP electroporation, Cas9 mRNA and sgRNA can also be delivered into cells *via* electroporation or liposome-based vectors (Evers et al., 2022; Yu et al., 2023) to improved genome editing fidelity. The rationale behind all these methods is to achieve a transient peak expression of CRISPR/Cas9 followed by quick turnover of these editors to avoid off-target effects due to prolonged editor expression.

The expression duration of genome editors also influences the choice of vectors for *in vivo* genome editing. For example, adeno-associated virus (AAV) and lipid nanoparticles (LNPs) are currently the primary vectors for *in vivo* gene therapy. AAV-delivered gene expression is known to last for years in terminally differentiated cell types (Lu et al., 2019). While this property might be favored in gene supplementation applications, AAV-mediated gene editing likely exhibits the propensity to accumulate undesired off-target mutations over time (Hanlon et al., 2019; Kim et al., 2019). By contrast, LNP-delivered Cas9 mRNA and sgRNA can be quickly degraded *in vivo*, thus LNP is currently the most popular vector for *in vivo* gene editing (Zuris et al., 2015; Wang et al., 2016b; Finn et al., 2018; Qiu et al., 2021; Ju et al., 2022; Ghani et al., 2023), which has already lead to investigational new drugs in clinical trials (NCT04601051) (Niemietz et al., 2020; Witzigmann et al., 2020; Gillmore et al., 2021) (Table 2).

## 5 Conclusive remarks

Efficiency and specificity are the two critical parameters that determine the success of CRISPR/Cas9-based editing of the genome. The tremendous potential of genome editing for gene therapy urges scientists to fully address its safety concerns, particularly the off-target effects. Methods to assess off-target effects of CRISPR/Cas9 have quickly evolved in the last decade. However, limitations still remain in balancing the accuracy versus sensitivity of these new techniques. Direct assessment of off-target effects *in vivo* and even in patients is particularly challenging. The development of solutions for these problems would give rise to next-generation genome editing tools that accelerate the arrival of the gene therapy era.
